# Non-interventional study to collect data for the application of lidocaine gel 2% during scaling and root planing and professional mechanical plaque removal

**DOI:** 10.1007/s00784-018-2468-0

**Published:** 2018-05-01

**Authors:** Gregor J. Petersilka, Nicole B. Arweiler, Joachim Otto, Tobias Wittig

**Affiliations:** 1 Private Practice, Wuerzburg, Germany; 2 Department of Periodontology, Philipps-University of Marburg, Marburg, Germany; 3 Medical-Scientific Department, Chemische Fabrik Kreussler & Co. GmbH, Rheingaustrasse 87-93, 65203 Wiesbaden, Germany

**Keywords:** Periodontitis, Topical anesthetic, Pain, Professional mechanical plaque removal, Scaling and root planing

## Abstract

**Objectives:**

Evaluation of the safety and efficacy of a topical lidocaine gel 2% (LG) during scaling and root planing (SRP) and professional mechanical plaque removal (PMPR).

**Materials and methods:**

The anesthetic effects as well as unwanted effects of LG prior to or during SRP and PMPR were evaluated in an observational, non-randomized, non-interventional study design. A total of 385 treatments were recorded in 68 study centers all over Germany. Rating of the anesthetic effect of LG by treating personnel and patients using a four-item verbal rating scale (VRS), tolerability, safety (adverse effects), and need for additional local injection anesthesia (ALI).

**Results:**

In SRP as well as in PMPR, application of LG allowed a sufficiently pain-free therapy in more than 90% of the patients as stated on the VRS (SRP: 97.8%, PMPR: 93.75%). Overall, ALI was needed in only 4.23% of the patients treated (SRP: 5.3%, PMPR: 2.62%). One adverse effect occurred within the observation.

**Conclusions:**

Application of LG may offer a safe and effective way to achieve pain-free therapy in periodontal patients.

**Clinical relevance:**

Patient compliance is key to the success of periodontal maintenance therapy. Effective and safe pain control during various kinds of periodontal therapy might increase patient compliance and therefore contribute to the long-term treatment success, among other factors. With regard to the patients observed in this study, 47% had previously received periodontal maintenance therapy and were therefore familiar with the treatment and the associated pain.

## Introduction

Severe periodontitis was the sixth most prevalent disease condition in the world in 2010, affecting 743 million people worldwide [1]. Even though the results of the Fifth German Oral Health Study [2] showed a significant decrease in periodontal disease in Germany, it is still widespread: 51.6% of the young adults (aged 35–44) and 64.6% of the younger elderly (aged 65 to 74) suffered from periodontitis. The Studies of Health in Pomerania (SHIP) also revealed an improvement of periodontal conditions together with an increasing number of present teeth, thereby implying a growing need for treatment of moderately diseased teeth [3]. Consensus reports of the 11th European Workshop on Periodontology have affirmed the necessity for supportive periodontal therapy regimens for periodontitis patients [4, 5]. The success of preventive measures and periodontal therapy depends on patient compliance among other factors.

Although there is usually no anesthesia given for PMPR, many patients prefer some kind of anesthesia even during supragingival tooth cleaning. Up to 30% of the patients undergoing PMPR require pain control [6]. Injection anesthesia is still the most frequent choice for non-surgical periodontal treatment. However, many patients are afraid of the pain associated with dental injections and do not like the prolonged numbness [7]. This might lead to a reduced compliance in periodontal maintenance, especially for pain-sensitive patients or patients with needle phobia, also called trypanophobia [8]. One of the results from an observational study in China was that comfort during treatment is an important factor influencing compliance of patients with chronic periodontitis [9].

Various studies have demonstrated that topical anesthetic gels are a well-accepted possible alternative to either injection anesthetics [10, 11] or placebo [12, 13] for patients who need pain control during scaling and root planing (SRP).

Therefore, the aim of this non-interventional observational study was to collect data, under real-life practice circumstances, about the use of lidocaine gel 2% (Dynexan Mundgel®, LG) as a topical anesthetic for non-surgical treatment of periodontal disease and for PMPR in dental practices all over Germany.

## Materials and methods

### Ethical considerations

This non-interventional observational study was conducted according to the recommendations from the German Health authorities (BfArM, Federal Institute for Drugs and Medical Devices and Paul-Ehrlich-Institute, Federal Institute for Vaccines and Biomedicines) [14] and according to the formal guidelines of the AKG Code of Conduct [15].

The Ethical Committee of the University of Muenster, Germany, was consulted (2015-385-f-S). All subjects gave informed written consent (data privacy statement) before taking part in this study.

### Study subjects

A total of 385 subjects were enrolled in 68 participating study centers. The subjects had to be at least 18 years old and requiring scaling and root planing, professional mechanical plaque removal, or a different treatment where LG could be applied as anesthetic. Exclusion criteria were pregnancy and lactation as well as the presence of any contraindications for treatment with LG such as allergy.

### Study medication and treatment

Lidocaine gel 2% as used in this observational study is a white topical anesthetic gel that has been commercially available as a medicinal product in Germany [Dynexan Mundgel®, Chemische Fabrik Kreussler & Co. GmbH] in tubes and cylindrical carpoules as an anesthetic for temporary, symptomatic treatment of pain at the oral mucosa, gingiva, and lips since 1976. For each participating patient, one treatment session was observed and documented. Prior to the treatment patient demographics, periodontal screening and recording codes (PSR) and the indication were recorded. LG was applied into periodontal pockets or to the sulcus with a blunt cannula. Whether LG was applied into all periodontal pockets at once prior to treatment or sequentially for each quadrant to be treated was recorded. The maximum anesthetic effect occurs approximately 45 to 60 s after application. Therefore, no explicit rules for retention time before starting any therapeutic intervention were given. Re-application in cases of insufficient pain control was explicitly possible and had to be documented. If sufficient pain control could not be achieved, an additional local injection anesthesia (ALI) could be given. The evaluations were performed immediately after completion of treatment. The treating personnel were asked to evaluate the anesthetic efficiency as well as the handling using grades from 1 to 6 (1 = very good, 2 = good, 3 = satisfactory, 4 = sufficient, 5 = inadequate, 6 = unsatisfactory). The subjects were asked to evaluate the pain during treatment using a verbal rating scale (VRS) with four categories: no pain, mostly pain free, sufficiently pain free, not sufficiently pain free. The subjects were also questioned about unwanted effects and whether they would choose LG for a similar future treatment.

### Statistics

Since the study was designed as an observational trial, no ex-ante calculations of power and sample size were possible. Standard descriptive methods were used to summarize the parameters studied. Statistical analysis was performed using SAS® Version 9.4.

## Results

### Study population characteristics

A total of 385 subjects were enrolled into this observational trial study between September 2015 and July 2016, among them 229 female (59.95%) and 153 (40.05%) male patients. The average age was 53.79 (± 14.21) years, the youngest patient was 20, the oldest 86 years old. The majority of the patients were between 50 and 70 years old (48.81%). Within the cohort, 29.21% of the patients were active smokers and 6.04% were diabetics.

Out of 382 patients, 179 individuals (46.86%) had previously received periodontal maintenance therapy (PMT), many of those (31.13%) over 2–5 years or longer.

The most frequent indications for the application of LG were professional mechanical plaque removal (PMPR, *n* = 198, 55.15%) and scaling and root planing (SRP, *n* = 137, 38.16%). Other indications given for at least five patients were measuring of pocket depth (*n* = 15, 4.18%), PMT (*n* = 8, 2.23%), cleaning of dental implants (*n* = 6, 1.67%), and calculus removal (*n* = 5, 1.39%).

Twelve patients had taken oral analgesics on the day of treatment, primarily Ibuprofen (*n* = 8 patients, 3.12%). The patient evaluation and the preference concerning anesthesia for future treatment were compared for the overall study population versus the patients who had not taken analgesics.

For 187 patients (53.28%), the anesthetic gel was applied to all periodontal pockets prior to treatment, whereas 164 patients (46.72%) were sequentially anesthetized in the quadrant that was about to be treated.

**Fig. 1:**
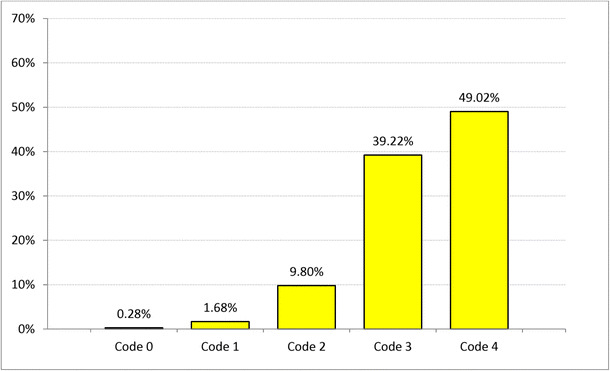
Frequency distribution in % of maximum PSR code for at least one sextant (n = 357)

**Fig. 2:**
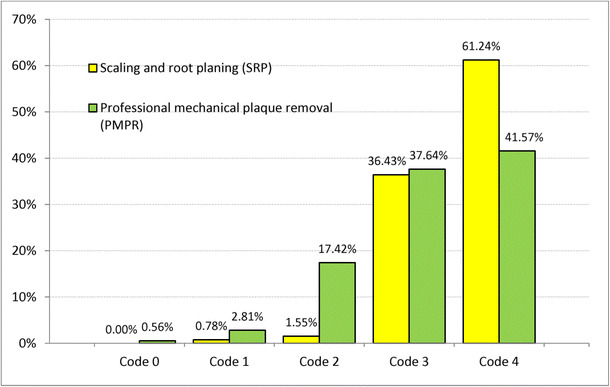
Frequency distribution of PSR codes by treatment categories

**Fig. 3:**
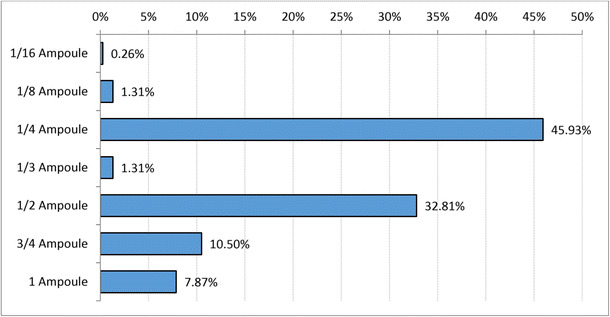
Amount of lidocaine gel 2% used (n = 381 therapies). One carpoule contains 1.7 ml gel with 34 mg of lidocaine

The periodontal screening and recording (PSR) code [16] was determined for the patients using the code given in Table 1. A total of 175 (49.02%) patients suffered from severe periodontitis in at least one sextant and 140 (39.22%) from moderate periodontitis (Fig. 1). Figure 2 compares the PSR codes for SRP and PMPR patients, showing that more patients in the SRP group suffered from severe periodontitis than in the PMPR group (61.24 vs. 41.57%); the incidence of moderate periodontitis, however, was not different in both groups. Figure 3 shows the amount of LG used. One cylinder carpoule with 1.7 g gel contains 34 mg lidocaine. The amount applied was determined by the treating personnel. According to the product guidelines (SmPC), the amount used should not exceed 40 mg of lidocaine per day.

**Table 1:** PSR code

Code	Clinical signs	
0	Absence of clinical signs	(healthy gingiva)
1	Bleeding on probing	(corresponds to gingivitis)
2	Supra and/or subgingival calculus and/or defective margins	(corresponds to gingivitis)
3	Periodontal pocket 4 to 5.5 mm deep	(corresponds to moderate periodontitis)
4	Periodontal pocket 6 mm deep	(corresponds to severe periodontitis)

### Efficacy

**Fig. 4:**
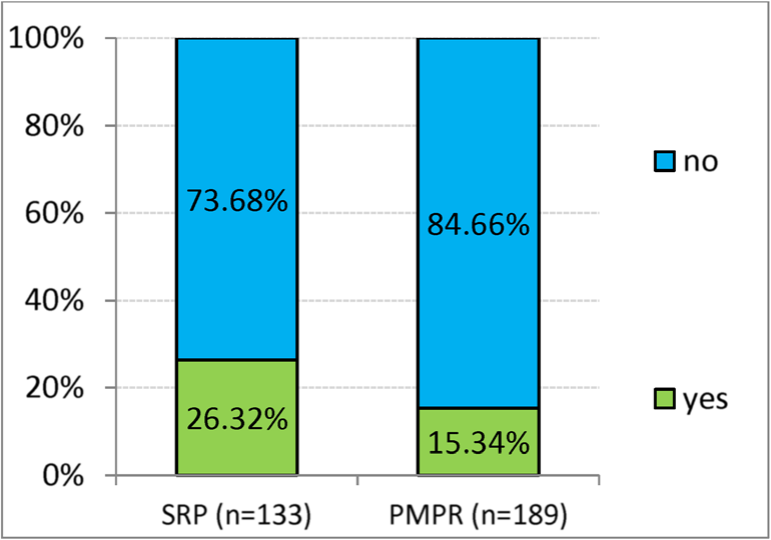
Percentage of requested re-application of lidocaine gel 2% for scaling and root planing (SRP) and professional mechanical plaque removal (PMPR)

Seventy-seven patients (20.42%) required a re-application of LG for single dental pockets or sulcus areas. The need for re-application was similar for patients who received full-mouth anesthesia prior to treatment (18.68%) and for patients who received sequential anesthesia per quadrant (23.31%). The values for SRP and PMPR patients differ as depicted in Fig. 4: 26.32% of the SRP patients required a re-application as opposed to only 15.34% of the PMPR patients. Additional injection anesthesia (“rescue anesthesia”) was needed by 16 patients, 4.23% of the study population. More SRP patients (5.3%) needed a rescue anesthesia than PMPR patients (2.62%). A total of 4.95% of the patients who received LG for all periodontal pockets/sulci at once needed ALI, as opposed to 3.07% of the patients who were anesthetized per quadrant.

### Evaluation by therapist

**Fig. 5:**
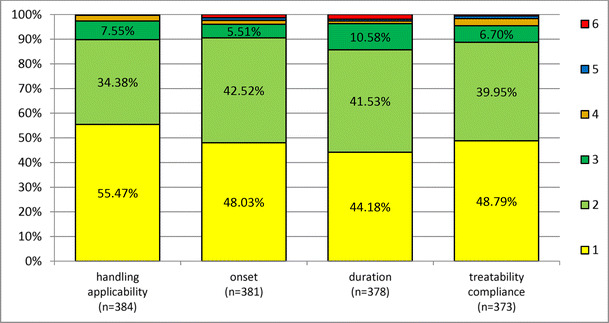
Evaluation of lidocaine gel 2% application by the therapists. Numbers from 1 to 6 indicate the perception of handling using a six grade scale (1 = very good, 2 = good, 3 = satisfying, 4 = sufficient, 5 = inadequate, 6 = unsatisfactory)

The treating personnel were asked to evaluate the following categories by using grades from 1 to 6 (1 = very good, 2 = good, 3 = satisfying, 4 = sufficient, 5 = inadequate, 6 = unsatisfactory): handling/application, onset of anesthetic effect, duration of anesthetic effect, and patient treatability/compliance. The ratings for all four categories were predominantly “very good” or “good”. On average, handling/application was rated 1.58 (± 0.75), onset 1.69 (± 0.90), duration 1.78 (± 0.96), and treatability/compliance 1.69 (± 0.87) (Fig. 5).

### Evaluation by patient

**Fig. 6:**
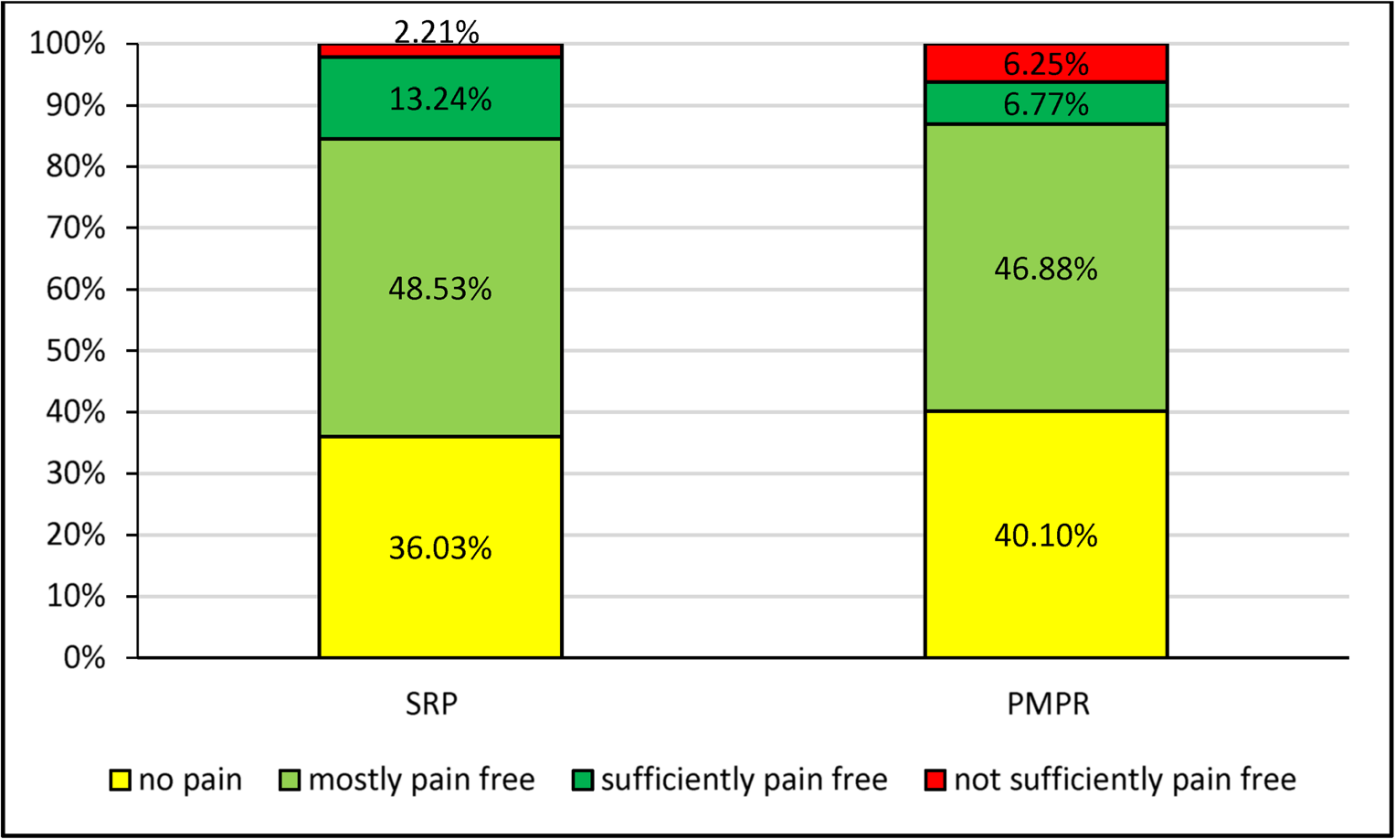
Patient evaluation of efficacy depicted separately for patients receiving scaling and root planing (SRP, *n* = 136) vs. professional mechanical plaque removal (PMPR, *n* = 192)

A total of 146 (38.02%) of 384 patients did not perceive any pain during the treatment, 179 (46.61%) were mostly pain free, and 40 (10.42%) were sufficiently pain free. Nineteen (4.95%) patients indicated that they were not sufficiently pain free. The average score was 1.82 (2 = mostly pain free). The evaluations by the sub-population of patients who had not taken any analgesics were comparable (37.90% no pain, 47.04% mostly pain free, 10.22% sufficiently pain free, and 4.84% not sufficiently pain free). Figure 6 depicts the patient evaluations for SRP and PMPR treatments, indicating rather small differences between both groups. Fewer patients in the SRP group were completely pain free (36.03%, *n* = 136) compared to the PMPR group (40.10%, *n* = 192), but the number of patients who were not sufficiently pain free was higher in the PMPR group (6.21%) compared to the SRP group (2.21%). A total of 350 (92.35%) of 379 patients would choose LG for a similar future treatment, whereas 29 (7.65%) patients would refuse a repeated LG application. The decision is the same for the 373 patients who had not taken analgesics on the treatment day (92.64% yes and 7.36% no). The preference for LG for a fictive future treatment was even stronger in the SRP group (96.95%, *n* = 131) as opposed to the PMPR group (90.22%, *n* = 184).

### Safety

Only one adverse effect was observed during this non-interventional study: one patient experienced an oral hypoesthesia (numb tongue) for about 15 min. No serious adverse effects were reported.

## Discussion

A major problem of studies assessing pain or pain levels is the fact that pain perception is subjective and may vary considerably between individuals. Aches and pains are considered complex products of a variety of physical, cognitive, and emotional factors. In a clinical setting as the one assessed here, the operator is an additional important factor, which may act as confounder, since dexterity and operation skills are important factors influencing the pain level perceived by the patient. Data indicates that correct communication and explanation about the topic of pain prior to therapy may lower the need for anesthetics [17]. These highly person-related factors are difficult to control even in a prospective randomized and controlled study setting. As opposed to randomized clinical trials that operate in an idealized environment with a pre-selected homogenous patient population, observational studies allow the collection of data from routine conditions in daily clinical practice [18, 19]. The aim of this observational study was to collect true-to-life data about the use of lidocaine gel 2% as a topical anesthetic in dental practices. The effect of the same anesthetic gel on pain sensitivity and wound healing, when applied following SRP, has been tested in a previous study [20]. In most cases observed in this study, the anesthetic gel was used for scaling and root planing and professional mechanical plaque removal and the overall acceptance by all patients was very high: 92% of the patients would choose LG for a similar future treatment. This clearly demonstrates the patients wish for some form of anesthesia not only for SRP but also for PMPR. A total of 38% of the patients reported no pain during treatment and another 47% were mostly pain free. Together with another 10% reporting being sufficiently pain free, about 95% of all patients were sufficiently anesthetized with LG. The average score given by the patients was 1.82 thus indicating a “mostly pain free” therapy. As expected, the pain perceived by patients undergoing SRP compared to that of patients receiving PMPR was slightly higher and so was the need for a re-application of LG with 26% for SRP and 15% for PMPR patients. However, the number of patients requiring re-application was considerably lower than that found in a randomized clinical trial (RCT) comparing injection anesthesia with two different anesthetic gels and placebo [21], where almost half of the patients needed re-application of the lidocaine/prilocaine gel for effective pain control during SRP. Nevertheless, the data collected in the current study stems from an observational design with a different investigational product and may therefore not be fully comparable to the results of Antoniazzi et al. [21].

If the choice for LG in a future treatment is regarded separately, the preference among the patients who received SRP is even higher with 97% than for the patients who received PMPR with 92%. The need for re-application seems to have no influence on the acceptance by the patients. This is consistent with studies comparing topical anesthetic gels with injection anesthesia which show a high preference for anesthetic gels by up to 80% of the patients [22, 23]. In an observational study, 72.4% of the patients preferred a lidocaine/prilocaine gel for SRP even though the treatment of deeper pockets caused increasing procedural pain levels [24]. Most likely, a certain amount of pain is accepted if an injection resulting in pain from the needle penetration as well as sustained numbness can be avoided.

The anesthetic depth that can be achieved with LG is sufficient for most patients, which can also be seen from the small percentage of patients, 5% for SRP versus 3% for PMPR, who actually needed “rescue anesthesia”. The evaluation of LG by the treating personnel with regard to the efficacy (onset and duration), handling, and patient treatability resulted in mostly “very good” and “good” grades, also demonstrating a very high acceptance. Whether freedom of pain is achieved by the use of LG or may simply be explained by the fact that the patient is less sensitive to pain cannot be assessed in an observational study. In addition, it can be assumed that subgingival debridement is perceived as being more unpleasant than supragingival cleaning. Another point to discuss is the impact of the method of instrumentation on perceived pain. Data indicates that patients may perceive hand instrumentation as being more painful than ultrasonic scaling even when topical anesthetics are applied [25]. However, data generated in a dental practice setting assessing the pain level during a routine scaling and polishing procedure revealed that without anesthesia, patients perceive curettes as less painful than ultrasonics [26]. It would have been interesting to stratify our data by instrumentation technique used; however, it was not documented, what type of instrument was used for debridement.

Only one adverse effect was observed in this non-interventional study, demonstrating a high degree of safety for LG. One patient suffered from a transitional numb tongue. It can only be speculated whether some gel was accidentally applied to the tongue or the patient moved his tongue to the gel applied to the sulcus. According to the PSR values recorded, 79% of the PMPR patients suffered from moderate or severe periodontitis, indicating that the PMPR performed could rather be classified as periodontal maintenance therapy.

## Conclusion

Lidocaine gel 2% offers a safe, effective, and highly accepted method of achieving a pain-free treatment for periodontal and maintenance patients.
